# A comparative study of a flow-cytometry-based assessment of *in vitro Plasmodium falciparum *drug sensitivity

**DOI:** 10.1186/1475-2875-8-294

**Published:** 2009-12-14

**Authors:** Stephan Karl, Rina PM Wong, Tim G St Pierre, Timothy ME Davis

**Affiliations:** 1 School of Physics, M013, The University of Western Australia, 35 Stirling Highway, Crawley WA 6009, Australia; 2 School of Medicine and Pharmacology, The University of Western Australia, Fremantle Hospital, Alma Street, Fremantle, WA, Australia

## Abstract

**Background:**

Recently developed Sybr Green-based *in vitro Plasmodium falciparum *drug sensitivity assays provide an attractive alternative to current manual and automated methods. The present study evaluated flow cytometry measurement of DNA staining with Sybr Green in comparison with the *P. falciparum *lactate dehydrogenase assay, the tritiated hypoxanthine incorporation assay, a previously described Sybr Green based plate reader assay and light microscopy.

**Methods:**

All assays were set up in standardized format in 96-well plates. The 50% inhibitory concentrations (IC_50_) of chloroquine, mefloquine and dihydroartemisinin against the laboratory adapted *P. falciparum *strains 3D7, E8B, W2mef and Dd2 were determined using each method.

**Results:**

The resolution achieved by flow cytometry allowed quantification of the increase in individual cell DNA content after an incubation period of only 24 h. Regression, and Bland and Altman analyses showed that the IC_50 _values determined using the flow cytometry assay after 24 h agreed well with those obtained using the hypoxanthine incorporation assay, the *P. falciparum *lactate dehydrogenase assay, the Sybr Green plate reader assay and light microscopy. However the values obtained with the flow cytometry assay after 48 h of incubation differed significantly from those obtained with the hypoxanthine incorporation assay, and the *P. falciparum *lactate dehydrogenase assay at low IC_50 _values, but agreed well with the Sybr Green plate reader assay and light microscopy.

**Conclusions:**

Although flow cytometric equipment is expensive, the necessary reagents are inexpensive, the procedure is simple and rapid, and the cell volume required is minimal. This should allow field studies using fingerprick sample volumes.

## Background

*In vitro *parasite drug susceptibility testing has an established role in both the evaluation of the prevalence of drug-resistant strains of *Plasmodium falciparum *in endemic areas [1-5] and screening novel compounds for anti-malarial activity [6,7]. Historically, the most widely used *in vitro *technique for assessment of drug resistance is the microscopic quantification of parasite maturation [8]. In this method, parasites are grown in serial drug dilutions in 96-well plates and cell suspensions from each well are used to prepare thick blood films. The number of schizonts is counted (usually per 200 parasites) after a maturation period of 20 to 40 h [9-12]. This approach is laborious, time consuming and unpopular with microscopists [13].

To circumvent these problems, light microscopic evaluation as the primary assay is increasingly replaced by new methods incorporating automated analysis of assay plates. The most widely used are i) quantitation of tritiated hypoxanthine incorporated into parasite DNA by scintillation counting [14], ii) colorimetric measurement of *Plasmodium *lactate dehydrogenase (LDH) [15,16], and iii) histidine rich protein II quantitation by Enzyme Linked Immunosorbent Assay [17]. Recently, the feasibility of using Sybr Green (SG) nucleic acid gel stain and fluorescence based analysis has been investigated [18-22]. In SG based assays, growth response is determined by detection of fluorescence using a photometric plate reader after a 48 h incubation period. Growth and multiplication of the parasites result in increased fluorescence signals from the control wells compared to those in the wells containing growth-inhibiting concentrations of a drug.

**Figure 1:**
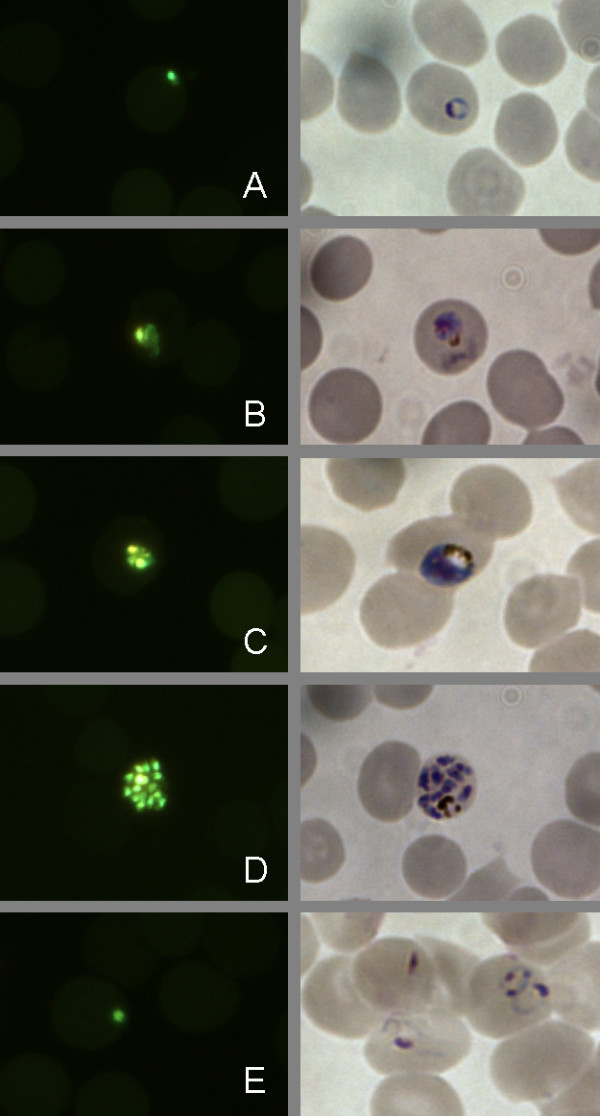
**Examples of *Plasmodium falciparum *parasites (strain Dd2) at 0 h (A) 12 h (B) 24 h (C) 36 h (D) and 48 h (E) after sorbitol synchronization under the fluorescence microscope after SG staining (left column) and in bright field (right column)**. The images were taken from corresponding slides prepared at the same time point from the same culture and prepared either for fluorescence microscopy or LM. Images were obtained on a Nikon Eclipse TE2000 -N Microscope with a 1000 × optical magnification with a Nikon LH-M100CB-1 Camera.

Over the course of the 48 h intra-erythrocytic life cycle of *P. falciparum*, the parasite develops from mono-nucleated ring forms into multi-nucleated schizonts (Figure 1). A schizont form is usually defined as an infected red cell containing three or more merozoites [12]. Each merozoite is packed with DNA and double-stranded DNA binding to SG will contribute to the fluorescence signal from an infected red cell. Fluorescence intensity is, therefore, a measure of parasite maturation to the schizont stage, a process which will be inhibited by therapeutic concentrations of anti-malarial drugs.

Here, a rapid and accurate *in vitro *assay system based on this principle is described, which employs a flow cytometer with attached high-throughput plate analyzer. The possibility of performing drug resistance measurements employing the technique of flow cytometry was first demonstrated by van Vianen *et al *in 1990 [23]. Since then, flow cytometers and software for analysis have improved considerably, allowing faster and less complicated assays based on flow cytometry. In the present study, a range of different automated and manual assays for drug sensitivity, namely the tritiated hypoxanthine incorporation assay (THA), the colorimetric measurement of *Plasmodium *lactate dehydrogenase (LDH), the Sybr Green plate reader assay (SGPR) and light microscopic (LM) cell count were compared with the flow cytometry based assay (FCMA). The assays were compared directly for up to four different parasite strains exposed to the anti-malarial drugs chloroquine (CQ), mefloquine (MQ) and dihydroartemisinin (DHA). The degree of agreement between the FCMA and the other four assays over a range of IC_50 _values was assessed using the methods of Bland and Altman [24,25]

## Methods

### Parasite culture

The laboratory-adapted *P. falciparum *strains 3D7, E8B and W2mef were cultured in RPMI 1640 HEPES (Sigma Aldrich, St Louis, MO) supplemented with 92.6 mg/L L-glutamine (Sigma Aldrich, St Louis, MO), 500 μg/L gentamicin (Sigma Aldrich, St Louis, MO), 50 mg/L hypoxanthine (Sigma Aldrich, St Louis, MO) and 10% v/v pooled human plasma (complete culture medium). Cultures were maintained with daily changes of medium at 5% haematocrit and diluted with red blood cells when the parasitaemia exceeded 5%. Cultures were incubated in an airtight desiccator cabinet at 37°C in an atmosphere containing between 5% to 10% oxygen. The low oxygen atmosphere was generated by gassing the cabinet with a mixture of 1% O_2 _and 5% CO_2 _balanced in N_2 _(BOC gases, Perth, Australia) at 1.0-1.5 bar for 60-90 s each time it had been opened.

The parasite strains 3D7, E8B and W2mef and Dd2 exhibit a range of drug sensitivities. 3D7 is sensitive to CQ and MQ. W2mef and Dd2 are MQ and CQ resistant. E8B is CQ resistant and MQ sensitive. All 4 strains are sensitive to DHA.

The drug sensitivity assays described below required exposure of parasites at the same stage of development after merozoite invasion. This synchronization was achieved by suspension of the cells in 5% w/v sorbitol (Sigma Aldrich, St Louis, MO) for 12 min to allow destruction of mature parasite stages through osmotic pressure change, followed by re-suspension of erythrocytes containing viable parasite forms in culture medium [26]. Such single-step sorbitol synchronization produces cultures that contain ring and early trophozoite stages that have developed for up to 18 h after merozoite invasion.

### Anti-malarial drugs

CQ diphosphate, MQ hydrochloride and DHA were obtained from Sigma Aldrich (St. Louis, MO). Stock solutions for CQ were prepared in distilled water, for MQ and DHA in 70% v/v ethanol and all were stored at -20°C. On the day of assay, aliquots were thawed and further diluted in RPMI to make a 5 μM working standard and subsequent two-fold serial dilutions in complete RPMI were performed. Final test concentrations ranged from 25 to 1600 nM for CQ, 0.78 to 200 nM for MQ and 0.1 to 51.2 nM for DHA. A set of drug dilutions was prepared in complete culture medium without hypoxanthine for use in the THA. Parasite growth response was assessed using the assays described in the following sections. The drug concentrations resulting in 50% inhibition of parasite growth (IC_50_) were determined by non-linear regression analysis of the data using GraphPad Prism 4.0 (GraphPad Software, CA).

### Tritiated hypoxanthine incorporation assay (THA)

Measurement of the tritium labelled hypoxanthine incorporated during parasite replication can be used to determine parasite drug susceptibility [27]. These isotopic assays were conducted at 2% parasitaemia and 1.5% haematocrit. Each test was performed in triplicate. To 90 μL red cell suspension was added 100 μL of drug-containing medium and 10 μL of a working solution (5 mg/mL) of tritium labelled hypoxanthine, resulting in a final activity of 0.5 μCi/well. After 48 h incubation, the plates were subjected to four freeze-thaw cycles to achieve complete cell lysis and then harvested onto 96-well glass-fibre filtermats (Perkin Elmer, Waltham, MA) using a Harvester 96 (Tomtec Incorporated, Hamden, CT). After air-drying, the filtermats were sealed in plastic envelopes with 4 mL of beta scintillant and counted on a 1450 Microbeta Plus liquid scintillation counter (Wallac, Turku, Finland). Scintillation counts from each well were used to derive dose response curves.

### *Plasmodium falciparum *lactate dehydrogenase assay (LDH)

Drug susceptibility assays were conducted at 1% parasitaemia and 1.5% haematocrit in 96 well plates with a final well volume of 200 μL. After 48 h incubation with drug dilutions, the plates were subjected to freeze-thaw cycles, as above. A modification of a *P. falciparum *LDH detection method was used to assess parasite growth [15,28]. The haemolysate was homogenized by repeated vigorous pipetting and 10 μL from each well were added to 200 μL of Malstat solution (Trisma base 1.21 g in 90 mL deionized water with pH adjusted to 9.1, 200 μL Triton X-100 (Merck, Victoria, Australia), 2 g lithium-L-lactate, 62 mg 3-acetyl-pyridine-adenine-dinucelotide (Sigma Aldrich, St. Louis, MO), 10 μL of nitro blue tetrazolium solution (10 mg/mL) (Sigma Aldrich, St. Louis, MO) and 10 μL diaphorase solution (10 mg/mL) (Sigma Aldrich, St. Louis, MO). The *P. falciparum *LDH reaction was allowed to proceed at room temperature for 45 to 90 min during which colour development in drug-free wells was assessed. Interference by air bubbles was circumvented by using a blow-dryer over the plate. Absorbance values in each well at 650 nm as measured with a standard fluorometer (Fluostar Optima, BMG Labtech, Offenburg, Germany) were used to derive dose response curves.

### Sybr Green Plate Reader Assay (SGPR)

The SGPR assay was a modification of the method described by Smilkstein *et al *[20]. After 48 h incubation, the supernatant from each well of an assay plate was removed (approximately 200 μL) and replaced by a red cell lysis buffer (8.26 g/L NH_4_Cl, 1 g/L KHCO_3_, 0.037 g/L EDTA, volume 200 μL/well), which also contained Sybr Green I at a 5× concentration. The plate was then incubated at 37°C for 20 min and total fluorescence from each well was measured on a standard fluorescence plate reader at 520 nm (Fluostar Optima, BMG Labtech, Offenburg, Germany). The gain of the instrument was set to a value of 1200. The plate was shaken in the instrument for 5 s before reading. The absolute fluorescence values of each well were used to generate dose response curves.

### Light microscopy (LM)

A thin blood film was prepared from every drug dilution on a well plate after an incubation time of 48 h. The films were air dried, fixed with methanol and stained with 5% Giemsa stain for 10 min. The number of parasites in 500 red blood cells was enumerated using standard LM techniques. The parasite counts were used to derive dose response curves.

### Determination of parasite nuclear division by flow cytometry

**Figure 2:**
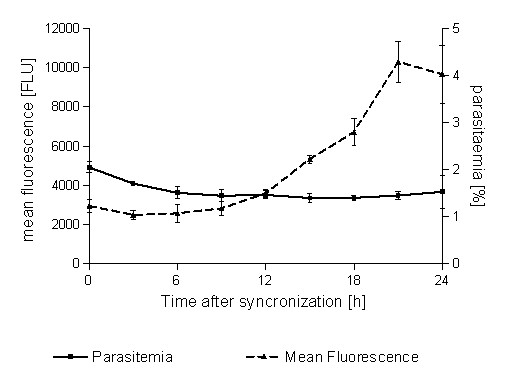
**Parasitaemia and mean fluorescence intensity in a synchronized parasite culture as observed with FCM at 3 h intervals over 24 h following synchronization (no drugs added)**. While parasitaemia remains stable, the mean fluorescence intensity increases markedly indicating schizont maturation. Fluorescence intensity is expressed in relative fluorescence intensity units (FLU).

**Figure 3:**
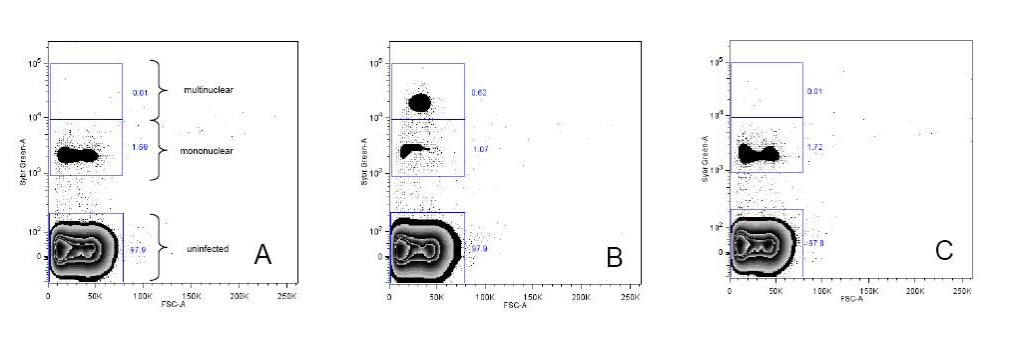
**Scatterplots of a parasite culture directly after synchronization (panel A), the same parasite culture after 24 h of incubation without drug (panel B) and the same parasite culture after 24 h of incubation with 1600 nM CQ (panel C)**. In drug-treated cultures, parasites do not progress to nuclear division and remain in the lower fluorescence intensity gate. Panel A also depicts the gating strategy used to distinguish between mononuclear and multinuclear parasitized cells in the FCMA. The number beside a gate denotes the percentage of the total events recorded in that gate.

Nuclear division in parasitized erythrocytes can be measured by flow cytometry. SG mainly stains the nuclei because they contain most of the DNA (Figure 1). A synchronized parasite culture was prepared for flow cytometric analysis (as described below) with sampling at the time of synchronization and at subsequent 3 h intervals over the next 24 h (see Figure 2). Based on the observations from this experiment, a gating procedure was developed which classified the cells as (1) uninfected, (2) infected mononuclear and (3) infected multinuclear. This gating strategy was subsequently applied in the FCMA (see Figure 3).

### Flow cytometric assay (FCMA)

Drug susceptibility tests were conducted at 1% parasitaemia and 1.5% haematocrit with a final volume of 200 μL per well and incubation at 37°C for a total of 48 h. After 24 h and 48 h, 10 μL of cell suspension was removed from each well and transferred into a new plate containing 180 μL PBS per well. A 10 μL aliquot of 1:100 SG was added to each well and the plates were incubated in the dark for 20 min. The plates were then centrifuged at 400*g for 5 min and the supernatant was replaced with 50 μL 1% w/v paraformaldehyde and 2.5% v/v glutaraldehyde in PBS for cell fixation. The plates were incubated for 60 min at 4°C, washed twice with 200 μL PBS and kept at 4°C before being read on a FACS Canto II Flow cytometer (BD Biosciences, Franklin Lakes, NJ) with attached plate coupler in high throughput mode. The flow cell on this instrument was a quartz cuvette. The light source was a 20 mW/488 nm solid state argon laser. At the flow cell the effective wattage of this light source is typically around 15 mW. The laser beam had an elliptical geometry with 9 μm and 65 μm being the short and long axes of the beam profile respectively. The instrument was equipped with photomultiplier tube detectors to detect wavelengths emitted from interaction with the the 488 nm laser in the ranges of 750-810 nm, 670-735 nm, 610-637 nm and 564-606 nm. SG has its emission maximum at 520 nm and, therefore, the 564-606 nm channel was used for detection.

Fifty thousand events were acquired from each well. The processing time for a 96-well plate was approximately 25 min. A scatter plot of forward scatter (FSC) versus fluorescence intensity detected in the 564-606 nm channel for each well was automatically generated by the FACSDiva software (BD Biosciences, Franklin Lakes, NJ) used to control the instrument. Gating was conducted using standardized procedures and the FlowJo 8.7 software (Tree Star, Ashley, OR). Tables were generated containing the fraction of the total number of events detected in each of the gates. The tabular data were further processed using a Microsoft Excel macro, which formatted the data for subsequent analysis with Graphpad Prism 4.0 (GraphPad Software Inc.).

Since the cell volume required for the FCMA is small, analyses at multiple time points are possible. Given the stage at which the cultures were synchronized, a further measurement after 48 h of incubation was made which reflected parasite multiplication rather than maturation. For the FCMA 24 h incubates, the response curves were generated using the number of events corresponding to multi-nuclear, schizont form parasites. For the 48 h incubates, the drug dose response curves used the total number of fluorescent events corresponding to all infected cells.

### Comparison of assays

All drug sensitivity tests were performed in triplicates except for the LM assay where only a single test was performed. Plates for all five methods were set up simultaneously from the same culture. One IC_50 _value was calculated from each triplicate. The total number of triplicate measurements (n) for each method/strain/drug combination was as detailed below, except for the LM assay where n corresponds to the number of single measurements.

#### FCMA 24 h versus LDH and THA

(CQ: 3D7 n = 4, E8B n = 2, W2mef n = 3, Dd2 n = 4);(MQ: 3D7 n = 3, E8B n = 2, W2mef n = 3, Dd2 n = 4);(DHA:3D7 n = 3, E8B n = 2, W2mef n = 3, Dd2 n = 4);

#### FCMA 48 h versus LDH and THA

(CQ: 3D7 n = 4, E8B n = 1, W2mef n = 3, Dd2 n = 4); (MQ: 3D7 n = 4, E8B n = 2, W2mef n = 3 Dd2 n = 4,);(DHA: 3D7 n = 4, E8B n = 1, W2mef n = 3, Dd2 n = 4);

#### Comparison between all assays (FCMA 24 h, FCMA 48 h, THA, LDH, SGPR, LM)

(CQ: 3D7 n = 2, W2mef n = 2, Dd2 n = 4); (MQ:3D7 n = 2, W2mef n = 2, Dd2 n = 4); (DHA: 3D7 n = 2, W2mef n = 2, Dd2 n = 4);

### Data analysis

**Figure 4:**
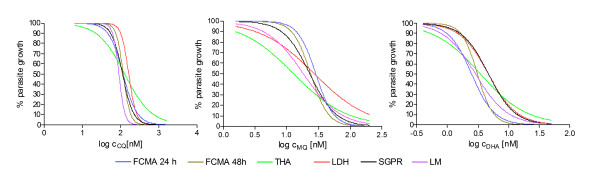
**Representative dose response curves for a set of parallel assays using the Dd2 strain**. **Panel A **shows the responses measured by the different methods to CQ, **Panel B **to MQ and **Panel C **to DHA.

Data from all five assays were analyzed using Prism 4.0 software (GraphPad Software Inc.). The datasets (scintillation counts for the THA, absorbance values for the LDH, fluorescent event counts for the FCMA, absolute fluorescence for the SGPR, and parasite counts for the LM) were normalized so that the smallest value in each dataset corresponded to 0% and the largest value corresponded to 100%. The normalized values were plotted over the decadic logarithmically transformed drug concentrations. Dose-response curves of the following form were fitted to the data:(1)

where *Y *corresponds to the percentage of growth at a drug concentration *X*, *k *is the Hill slope, and *IC*_50 _is the drug concentration causing a 50% reduction in *Y*. An example for sets of dose response curves obtained from one parallel assay setup for the Dd2 strain can be found in Figure 4.

Pair-wise correlations between the FCMA assay and the other four assays were assessed using orthogonal linear regression analyses (Deming model) to account for the variability in both *x *(Method A) and *y *(Method B). Agreement between methods was further evaluated by the Bland-Altman method [24,25].

## Results

### Determination of parasite nuclear division and FCMA development

As in the microscopy-based schizont maturation assay, the FCMA can enumerate parasites which have matured after a period of time not exceeding the full intra-erythrocytic life cycle. In the present study, an incubation time of 24 h was used. Figure 1 illustrates the principle of flow cytometric determination of parasite maturation based on parasite nuclear division in infected erythrocytes in a synchronized parasite culture. While there was no marked increase in parasitaemia (number of recorded events in the region corresponding to infected cells) over the 24 h following synchronization, the mean fluorescence intensity of the parasitized fraction of cells increased substantially, consistent with parasite maturation (Figure 2). Events corresponding to parasitized erythrocytes fell either into a low fluorescence intensity gate or a high fluorescence intensity gate depending on the fluorescence intensity of the DNA content. The number of events recorded in the high fluorescence intensity gate after an incubation time of 24 h corresponds to the number of multinucleated schizonts, which have developed over that time.

The scatterplots in Figure 3 show an initially synchronized parasite culture of clone 3D7 (panel A), the same parasite culture after 24 h without drug (panel B), and the same parasite culture after 24 h incubation with an inhibitory concentration of CQ (panel C). The untreated parasite culture developed during incubation, resulting in a higher percentage of infected cells in the high fluorescence intensity gate. By contrast, the parasites in the culture containing CQ did not develop and the vast majority of recorded events corresponding to infected cells remained in the low fluorescence intensity gate. When incubating parasites with different concentrations of drug, the percentage of cells recorded in the high fluorescence intensity gate is, therefore, a measure of drug action and batch processing of the scatterplots obtained from a well plate analysis can be used to generate drug response profiles.

Normal development of synchronized *P. falciparum *results in stepwise multiplication at 48 h intervals. Therefore, the number of infected cells increases without drug pressure but remains the same when exposed to inhibitory drug concentrations. Thus the scatterplot after 48 h of incubation yields information regarding the effect of the drug on parasite multiplication.

### Comparison of FCMA, THA and LDH, SGPR and LM

**Figure 5:**
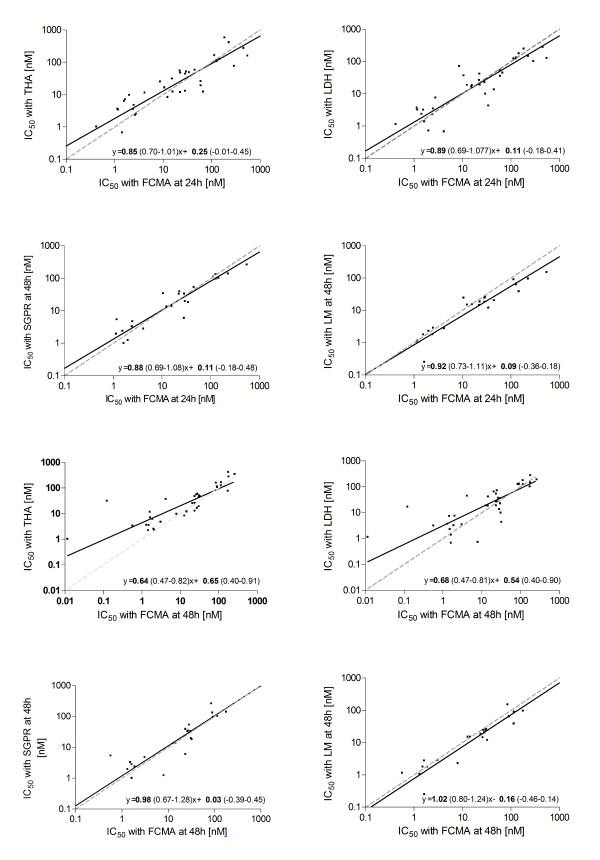
**Orthogonal linear regression (Deming model) for pair-wise comparison of the different assays**. **Panel A**: FCMA at 24 h and THA, **Panel B**: FCMA at 24 h and LDH, **Panel C**: FCMA at 24 h and SGPR, **Panel D**: FCMA at 24 h and LM. **Panel E**: FCMA at 48 h and THA, **Panel F**: FCMA at 48 h and LDH, **Panel G**: FCMA at 48 h and SGPR, **Panel H**: FCMA at 48 h and LM The solid black lines are the best fit lines of the regression. The dashed grey lines represent the line of identity. Regression equations are given with each plot together with the 95% confidence intervals on the fitted parameters.

**Figure 6:**
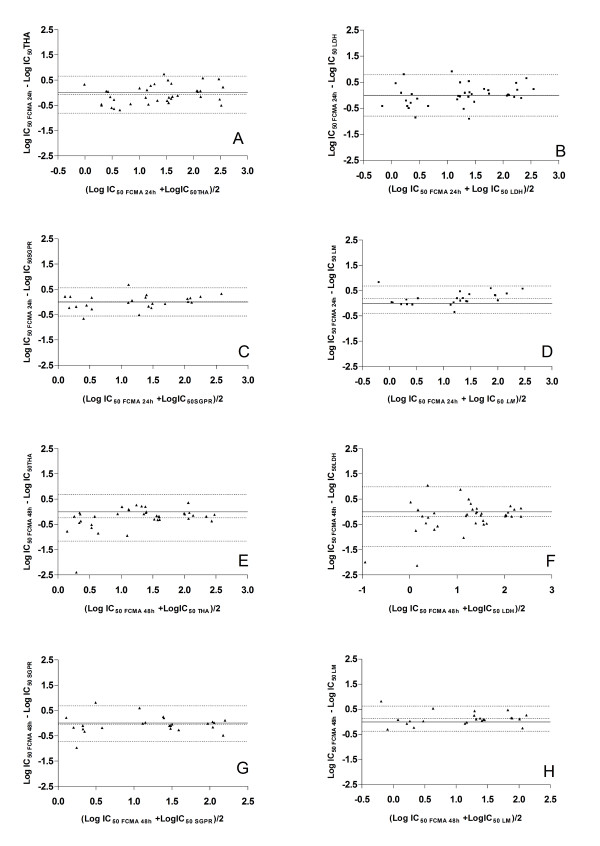
**Bland Altman plots displaying the differences between individual measurements of IC_50 _(as described in the methods section) using different pairs of techniques**. **Panel A**: FCMA at 24 h and THA, **Panel B**: FCMA at 24 h and LDH, **Panel C**: FCMA at 24 h and SGPR, **Panel D**: FCMA at 24 h and LM, **Panel E**: FCMA at 48 h and THA, **Panel F**: FCMA at 48 h and LDH, **Panel G**: FCMA at 48 h and SGPR, **Panel H**: FCMA at 48 h and LM. The differences in logarithmically transformed IC_50 _values for a paired measurement are plotted over the logarithmically transformed averages of the paired measurements. The inner dotted line is the average difference of the IC_50 _values while the outer dotted lines denote the upper and lower 95% limits of agreement between the two techniques.

The differences in absolute IC_50 _values between methods were not normally distributed (as assessed by the Kolmogorov-Smirnoff test), but logarithmic transformation produced values that were normally distributed about their mean. Therefore, for orthogonal linear regression and Bland-Altman analysis the logarithms of the IC_50 _values were used. Figure 5 shows the results of the orthogonal linear regression analysis of pair-wise comparisons of the IC_50 _measurements obtained from the FCMA assays with those from the other four assays. Results obtained using the Bland-Altman method are shown in Table 1 and Figure 6. All assays produced similar IC_50 _values after 24 h of incubation over the full concentration range used in the present study. The FCMA-derived IC_50 _values at 48 h tended to be lower than those of the THA and LDH at drug concentrations <10 nM. However the FCMA at 48 h corresponded well with the SGPR and LM methods.

**Table 1:** Geometric means and (lower and upper 95% limits of agreement) in log [nM] for the differences in IC_50_s between the methods

	FCMA (48 h)	FCMA(24 h)	THA	LDH	SGPR
**FCMA (24 h)**	0.18 (-0.79, 1.15)	-	-	-	-
**THA**	-0.24 (-1.16, 0.68)	-0.07 (-0.80, 0.66)	-	-	-
**LDH**	-0.19(-1.38, 0.99)	0.003 (-0.80, 0.79)	0.07 (-0.85, 1.00)	-	-
**SGPR**	-0.04 (-0.72, 0.68)	0.01 (-.0.55, 0.56)	-0.18 (-0.81, 0.45)	0.08 (-0.51, 0.67)	-
**LM**	0.13 (-0.37, 0.63)	0.18 (-0.33, 0.68)	-0.36 (-1.06, 0.35)	-0.09 (-0.77, 0.58)	0.17 (-0.33, 0.68)

## Discussion

Based on regression and Bland-Altman analyses, the FMCA performed well when compared with the other four *in vitro *assays of *P. falciparum *drug sensitivity, especially when restricted to a 24 h incubation. Parasite forms in the second half of the life cycle are not seen in blood taken from patients with falciparum malaria since erythrocytes containing these mature parasite forms cytoadhere within the microvasculature. Thus, prior synchronization of field samples appears unnecessary.

The discrepancies between FMCA at 48 h and the THA and LDH at low drug concentrations may relate to the nature of the assays. Whereas the LDH and THA measure parasite metabolic activity on a continuous scale, the fluorescence based assays measure parasite metabolic activity 'step-wise' with the signal increasing only if new nuclei are formed. It may be that lower drug concentrations still allow a degree of parasite metabolic activity, which is not measurable with fluorescence based assays because it does not result in a nuclear division.

The FCMA has several advantages over the other methods. Although the flow cytometric equipment is expensive and available only in specialized centres, the necessary reagents are inexpensive (approximate cost US$1.50/96 well plate). Accurate results from FCMA were obtained after an incubation time of 24 h. This contrasts with the usual 42 h used with THA assays [4,14,29-31], and 48 h for both the LDH and other assays based on SG [15,20-22]. The LM assay can be evaluated after 24 h incubation. However, LM is very labour-intensive.

After incubation, processing and flow cytometry can also be performed rapidly in the FCMA technique, with the high-throughput coupler being able to process a 96 well plate in about 25 min. By comparison, the assay as described by van Vianen 1990 [23] required 2 h for plate reading. In the present study, cells were fixed, which requires additional washing and incubation steps. However, once the cells are fixed, the plates can be kept for up to several weeks before being analysed. In laboratories where cell fixation is not required prior to flow cytometry, addition of the fluorescent dye, a short incubation period and subsequent flow cytometry may be sufficient to obtain valid results, reducing the processing time to a few minutes.

**Figure 7:**
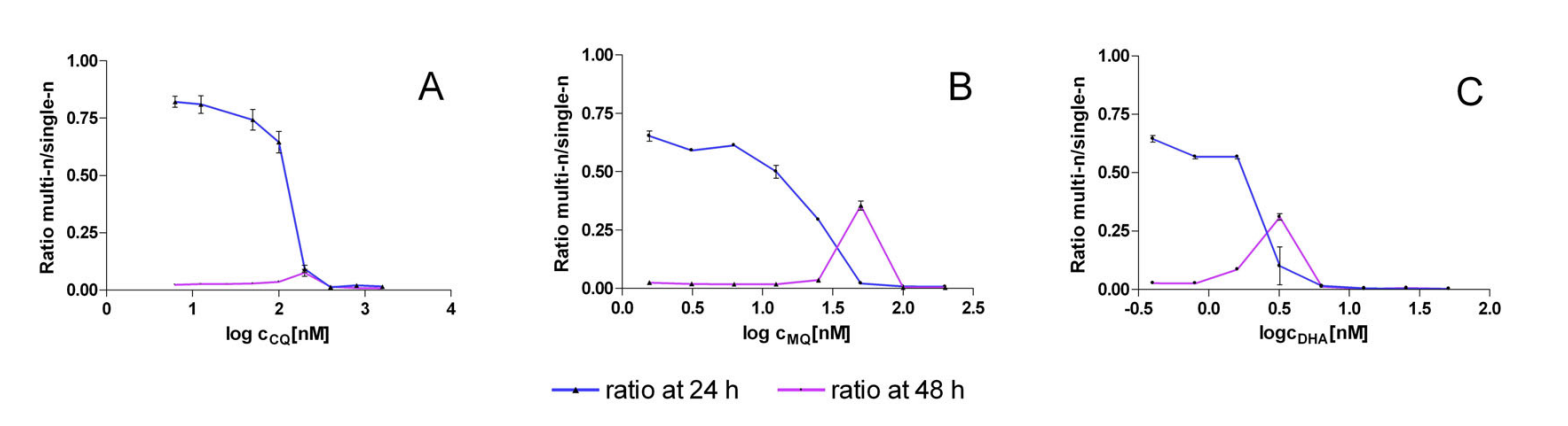
**Ratio of multi nucleated (multi-n) parasite forms to single nucleated (single-n) parasite forms after an incubation time of 24 h and 48 h for a representative experiment with the Dd2 strain (Panel A for CQ, Panel B for MQ and Panel C for DHA)**. The drug concentrations which inhibited parasite development into multi-n forms after 24 h still allowed maturation of some parasites after 48 h especially for the drugs MQ and DHA.

Another advantage of the present FCMA method is that it allows a basic assessment of stage specific drug sensitivity. Although it can only distinguish between parasites containing a single nucleus and multiple nuclei, the present data show that development of the parasites is slowed but not stopped at certain drug concentrations. While this effect was not seen at 24 h, it became evident at 48 h using the FCMA. Parasites exposed to drug concentrations at least twice those which inhibited reinvasion still developed into multi nucleated forms in 48 h. The delay in parasite maturation becomes evident when looking at the ratio between single nucleated and multinucleated parasite forms in the test wells as shown in Figure 7.

A further advantage is the relatively small number of cells required for the FCMA. In the present study, FCMA plates were set up at 1.5% haematocrit and small volumes (10 μL/well) were removed from the original plates and suspended in 190 μL/well PBS in new 96 well plates. This procedure resulted in a final cell concentration of about 7.5 × 10^3 ^μL^-1^. This enables serial FCM analyses from the same 96 well plate, for example where duration of drug exposure is of interest. With the very low cell number required, it is possible to set up the FCMA using a very low initial haematocrit so that the volume of blood obtainable from a fingerprick would be enough to set up an entire 96-well plate.

There are potential sources of interference with the FCMA system. Although white blood cells in an unprocessed fingerprick sample exhibit much higher fluorescence than malaria parasites, they can easily be gated out. In the present study, the culture medium was supplemented with human serum that caused some background noise in the flow cytometric measurements. However, if human serum is substituted by Albumax II (Invitrogen, Carlsbad, CA) this can be completely abrogated.

Several assays have been developed, many of them very recently, employing flow cytometry to measure parasite drug resistance[23,32-35]. The FCMA presented here has advantages when compared with all these assays. All but one of the previous studies have used 42 h-48 h incubation periods. Also, only a few of these studies used a high throughput flow cytometer. The present assay combines the advantages of previous assays. It uses a simple, inexpensive single staining procedure without cell lysis, and allows stage specific discrimination of malaria parasites and high throughput measurement after short incubation time (24 h). The present study is the first to compare flow cytometric measurement of parasite maturation after 24 h with four established reference methods.

Recently, Izumiyama *et al *[33] presented preliminary data on an FCM assay measuring drug resistance after 25 h of incubation using Sybr Green. The study was focused on the basic principles of stage discrimination by Sybr Green based on flow cytometry but did not use high throughput flow cytometry. Even more recently, a study by Grimberg *et al *[32] used a sophisticated well plate based flow cytometry assay with a combination of dyes to discriminate between DNA and RNA and determine life cycle stages. While Grimberg's method may allow for more differential stage discrimination, since RNA was measured as a separate parameter, this method is more complicated, and requires more expensive equipment such as a multi-laser flow cytometer with at least one laser in the UV range.

## Conclusion

Flow cytometry based measurement of fluorescence from SG stained cell samples provides a method of rapidly and accurately analyzing the drug sensitivity of *P. falciparum in vitro*. Although not yet available in many laboratories in developing countries, the technology may become more affordable in the near future. The relative ease of the assay, its potentially high throughput and the low reagent costs once the system is established could enable large-scale assessment of local parasite resistance patterns as well as facilitate screening of much-needed new anti-malarial compounds.

## List of abbreviations

CQ: chloroquine; DHA: dihydroartemisinin; DNA: deoxyribonucleic acid; FCMA: flow cytometric assay; IC_50_: 50% inhibitory concentration; LM: light microscopy; MQ: mefloquine; LDH: *Plasmodium falciparum *lactate dehydrogenase assay; SG: Sybr Green; SGPR: Sybr Green plate reader assay; THA: tritiated hypoxanthine incorporation assay.

## Competing interests

The authors declare that they have no competing interests.

## Authors' contributions

SK conceived the study and developed the FCMA. RPMW and SK conducted experimental procedures, data collection and analysis. SK, RPMW, TSP, TMED interpreted the data and wrote the manuscript. All authors read and approved the final manuscript.
